# Comparative Expenditure in Hospitals

**Published:** 1888-04-21

**Authors:** 


### COMPARATIVE EXPENDITURE IN HOSPITALS.

I can hardly agree to your criticism on the diet-table of a Northern Hospital. I think English people generally are mistaken as to the quantity and quality of food necessary for health. Of course labouring men require more food than those leading a sedentary life, but that increase should be in the fatty and starchy material. I am sixty-one; height, 5 ft. 9 in.; weight, 132 lbs. Here is my diet: Rise at 6.30, and while dressing take 8 oz. of warm milk. Breakfast, 8.30 a.m.; 3 oz. oatmeal made into porridge, with 8 to 10 oz. milk; a bowl of milk curdled with rennet—about 8 oz. Dinner at 1.30 p.m.; 8 oz. of milk, and the cup filled with coffee; bread, with about 1 oz. cold fat of ham; a bowl of curd same as breakfast. Tea at 6 p.m.; 10 oz. milk, with bread and preserve. Supper; three small biscuits. I have adopted this diet for more than twelve months in consequence of severe attacks of stomach and liver disorder, with hæmatemesis. I have fair health, can walk three miles at a stretch, or dig two hours in the garden, and I have abundance of mental energy. I eat about 1 lb. of bread in the day. The total food is less than 3 pints of milk, 10 oz. meal and flour in porridge and bread, and 1 oz. fat. This is far below the diet you criticise, yet I find it perfectly sufficient, and it leads me to think that there is great misapprehension as to the amount of food required, at least in the later portion of life.

An Old Physician.

P.S.—Before anyone criticises this letter, I must ask him to try the diet for three months, and report his weight at the beginning and the end. He may increase the quantity, but he must stick to the articles.

---

### NOTICE TO CORRESPONDENTS.

*R. S.*—All communications must be authenticated by writer's name and address, *not necessarily for publication.*

